# *FOXC2* Mutations in Familial and Sporadic Spinal Extradural Arachnoid Cyst

**DOI:** 10.1371/journal.pone.0080548

**Published:** 2013-11-22

**Authors:** Yoji Ogura, Shoji Yabuki, Aritoshi Iida, Ikuyo Kou, Masahiro Nakajima, Hiroki Kano, Masaaki Shiina, Shinichi Kikuchi, Yoshiaki Toyama, Kazuhiro Ogata, Masaya Nakamura, Morio Matsumoto, Shiro Ikegawa

**Affiliations:** 1 Laboratory of Bone and Joint Diseases, Center for Integrative Medical Sciences, RIKEN, Tokyo, Japan; 2 Department of Orthopaedic Surgery, Fukushima Medical University, Fukushima, Japan; 3 Department of Orthopaedic Surgery, School of Medicine, Keio University, Tokyo, Japan; 4 Department of Biochemistry, Yokohama City University Graduate School of Medicine, Yokohama, Japan; McGill University, Canada

## Abstract

Spinal extradural arachnoid cyst (SEDAC) is a cyst in the spinal canal that protrudes into the epidural space from a defect in the dura mater. Most cases are sporadic; however, three familial SEDAC cases have been reported, suggesting genetic etiological factors. All familial cases are associated with lymphedema-distichiasis syndrome (LDS), whose causal gene is *FOXC2*. However, *FOXC2* mutation analysis has been performed in only 1 family, and no mutation analysis has been performed on sporadic (non-familial) SEDACs. We recruited 17 SEDAC subjects consisting of 2 familial and 7 sporadic cases and examined *FOXC2* mutations by Sanger sequencing and structural abnormalities by TaqMan copy number assay. We identified 2 novel *FOXC2* mutations in 2 familial cases. Incomplete LDS penetrance was noted in both families. Four subjects presented with SEDACs only. Thus, SEDAC caused by the heterozygous *FOXC2* loss-of-function mutation should be considered a feature of LDS, although it often manifests as the sole symptom. Seven sporadic SEDAC subjects had no *FOXC2* mutations, no symptoms of LDS, and showed differing clinical characteristics from those who had *FOXC2* mutations, suggesting that other gene(s) besides *FOXC2* are likely to be involved in SEDAC.

## Introduction

**Figure 1:**
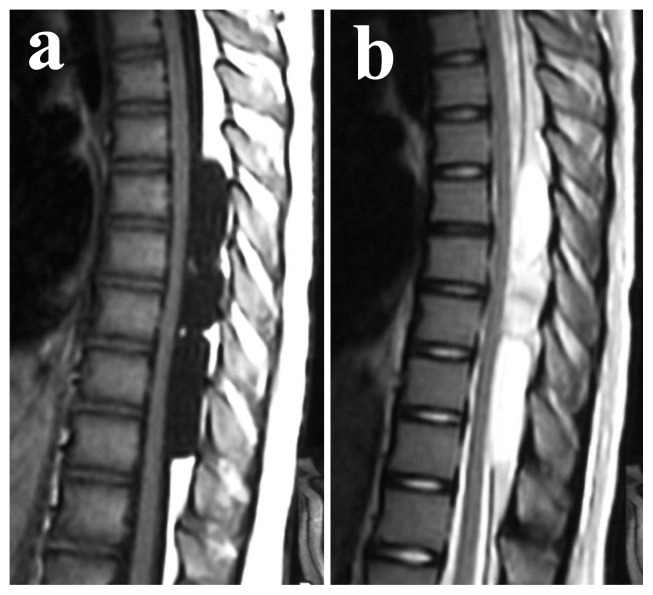
Spinal extradural arachnoid cyst. T1- (a) and T2- (b) weighted sagittal plane images of MRI (magnetic resonance imaging) scan. Subject III-2 of Family 1, 13 years old. There are multiple cysts dorsal to the spinal cord at the thoracolumbar spine.

Spinal extradural arachnoid cyst (SEDAC) is a cyst in the spinal canal that protrudes into the epidural space via defects in the dura mater (**Fig. 1**). It commonly occurs in the posterior thoracic area,[1] predominately affects males,[2] and is relatively rare, representing only 1% of all primary spinal tumors.[3] The cyst expands due to retention of cerebrospinal fluid that collects via a pedicle connecting the intra- and epi-dural subarachnoid spaces, in response to changes in spinal pressure. An expanding cyst may compress the spinal cord and cause neurological disturbances.[4] SEDAC is surgically curable; however, early diagnosis is important because delayed treatment leads to irreversible neurological defects.[4]

The etiological factors of SEDAC remain unclear. Its origin has been attributed to congenital dural defects, arachnoid proliferation and inflammation, previous surgery, and closed spinal trauma.[5] A few reports have suggested genetic etiological factors, since 3 families with SEDAC have been reported, including a pair of siblings,[6] 3 siblings,[7] and a large pedigree.[8] Some members from the 3 families showed coexisting lymphedema in their lower extremities and distichiasis (double rows of eyelashes arising from the Meibomian glands).[6]–[8] These observations suggest that SEDAC is associated with lymphedema-distichiasis syndrome (LDS) (OMIM 153400).[7]–[9]

LDS is an autosomal dominant disorder with variable expressivity. Its major features are lymphedema and distichiasis. The penetrance of lymphedema or distichiasis is 70% to 80%.[9] Its minor features include ptosis, cleft palate, renal abnormalities, congenital heart disease, vertebral anomalies, and SEDAC.[8], [10]–[13] The minor features have lower penetrance and their details are unclear. The causal gene of LDS is *FOXC2*, a forkhead family transcription factor (OMIM 153400); in fact, molecular screening of 81 probands resulted in the detection of *FOXC2* mutations in 100% of LDS patients.[8], [9], [14]–[17] Therefore, *FOXC2* is a good candidate gene for SEDAC; however, *FOXC2* mutation analysis has been performed in only 1 SEDAC family associated with LDS, and no mutation analysis has been performed on sporadic SEDACs or SEDACs unrelated to LDS (solitary SEDACs). The relationship between SEDAC and *FOXC2* mutations remains unclear.

To gain insight into the genetic etiology of SEDAC, we examined *FOXC2* mutations in 2 familial and 7 sporadic cases of SEDAC.

## Materials and Methods

### Ethics statement

The study was approved by the institutional review boards of RIKEN Center for Integrative Medical Sciences, Keio University and Fukushima Medical University. A written informed consent was obtained from all participants and/or guardians on the behalf of the minors/children participants.

### Subjects

**Figure 2:**
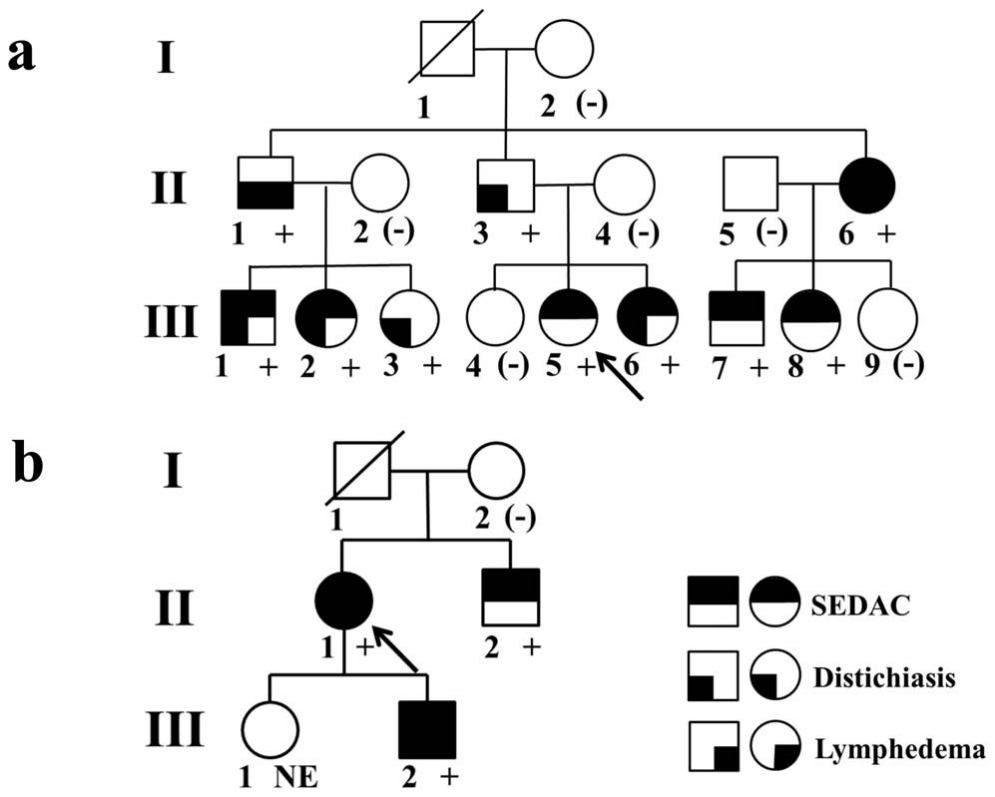
Pedigrees of the families with spinal extradural arachnoid cyst and co-segregation of the *FOXC2* mutations. a) Family 1 with c.733delG, b) Family 2 with c.354C>G. Some family members had distichiasis and/or lymphedema. Autosomal dominant mode of inheritance is definite when the disorder of the pedigree was considered as a syndrome consisting of spinal extradural arachnoid cyst, distichiasis and lymphedema. All affected subjects of the syndrome had heterozygous c.733delG or c.354C>G mutation in *FOXC2*. + and (-) indicate subjects with and without the mutations, respectively. NE indicates a subject who was not examined.

We recruited a total of 17 Japanese SEDAC subjects. Seven of them were from a previously reported family[3] (Family 1; **Fig. 2a**), three were from another family (Family 2; **Fig. 2b**) and 7 were sporadic SEDAC cases with no family history. All subjects had no history of infection, trauma and previous surgery of the spine. All but one proband had received surgery for SEDAC. There were no operative findings suggestive of infection and trauma. Ten subjects without SEDAC from the familial SEDAC pedigrees were also recruited for the DNA analysis. Magnetic resonance imaging (MRI) scans of the thoracic and lumbar spines were obtained for all subjects. The T1- and T2-weighted images in the sagittal plane were used for evaluation of SEDACs (**Fig. 1**).

### Mutation detection

DNA was extracted from saliva using the Oragene DNA self-collection kit (DNA Genotek, Ottawa, Canada) according to the manufacturer's protocol. The single coding exon of *FOXC2* (NM_005251.2) was PCR-amplified using KOD FX (TOYOBO, Osaka, Japan) and the primers 5′-TCTCCCCCTCTGGCTCTCT-3′ (forward) and 5′-TCTGCAGCCCCTTAATTGTC-3′ (reverse). PCR products were sequenced by the dideoxy termination method, using an ABI 3730 automated sequencer (Applied Biosystems, Foster City, USA), and screened for mutations.

### *In silico* structural analysis

To evaluate the effect of the N118K *FOXC2* variant on its ability to bind DNA, we used FoldX as previously described.[18] FoldX is an algorithm that calculates the free energy of proteins and nucleic acids based on a high-resolution 3D model of their structure, in order to predict the effect of mutations on their stability, folding, and dynamics. Because the structure of FOXC2 has not been identified, we analysed the FOXK2 protein, since both have similar primary sequences in their forkhead DNA binding domains.

### Quantitative PCR

The TaqMan real-time quantitative PCR (qPCR) method was used to examine the copy number of *FOXC2*. The primers and probes for the qPCR were designed using Primer Express software v3.0 (Applied Biosystems). The RNase P gene was used as a reference gene. All assays were performed with the TaqMan Universal PCR Master Mix according to the manufacturer's protocol (Applied Biosystems).

### Plasmid construction for western blot and the luciferase assay

The wild-type, c.354C>G, and c.733delG *FOXC2* genes were PCR-amplified using the primers 5′-TATGAATTCAATGCAGGCGCGCTACTC-3′ (forward) and 5′- TATAGATCTTCAGTATTTCGTGCAGTCGTAG-3′ (reverse). The PCR products were cloned into the *Eco*RI and *Bgl*II sites of the pFLAG-CMV-4 expression vector (Sigma Aldrich, St. Louis, USA). The nucleotide sequences of the clones were confirmed by Sanger sequencing.

### Cell culture and transfection

COS-7 and HeLa cells were grown in Dulbecco's modified Eagle's medium and 10% foetal bovine serum. Transfection was performed using FuGENE6 transfection reagent (Promega, Fitchburg, USA) according to the manufacturer's protocol. For protein expression, COS-7 cells were transfected in a 12-well plate using 500 ng of the FOXC2-pFLAG construct. For luciferase assays, HeLa cells were transfected in a 24-well plate using 500 ng of the FOXC2- pFLAG construct, 50 ng of the FOXC2 luciferase reporter (a gift from M. Walter), and 1 ng of the pRLTK control vector (Promega).[19] Transfected cells were grown for 48 h under 5% CO_2_ at 37°C.

### Western blot analysis

Transfected COS-7 cells were washed with PBS and harvested by scraping after 48 h of incubation. The cell lysates were resolved by SDS-PAGE. The N-terminal FLAG epitope was detected by immunoblot analysis, using a mouse anti-FLAG monoclonal antibody (Sigma Aldrich).

### Luciferase assay

Transfected HeLa cells were washed with PBS and dissolved in 150 ml of passive lysis buffer after 48 h of incubation. The dual-luciferase assays were performed in triplicate, using the Promega Dual Luciferase Assay kit (Promega), according to the manufacturer's protocol.

## Results

### Mutation analysis of *FOXC2*

**Figure 3:**
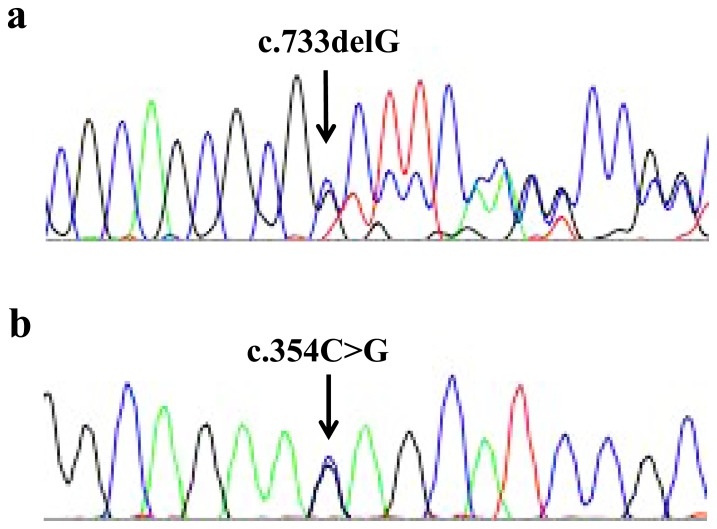
The *FOXC2* mutations in spinal extradural arachnoid cyst. a) A heterozygous deletion (c.733delG) in Family 1. b) A heterozygous missense mutation (c.354C>G) in Family 2.

We performed direct sequencing to analyse the PCR products of DNA obtained from familial and sporadic SEDAC subjects. We found 2 novel *FOXC2* mutations in 2 familial cases. In Familiy 1 (**Fig. 2a**), all the affected subjects had a heterozygous guanine deletion (c.733delG) (**Fig. 3a**). This mutation leads to an early stop codon at amino acid 277 (p.A245Pfs*32), which is unlikely to cause nonsense-mediated mRNA decay, since *FOXC2* is a single exon gene. This mutation was not found in any database or in the general population of 100 randomly sampled Japanese people. Furthermore, no *FOXC2* mutations were found in 6 subjects who belonged to the family and did not have any symptoms of SEDAC, distichiasis, or lymphedema (**Fig. 2a**).

**Figure 4:**
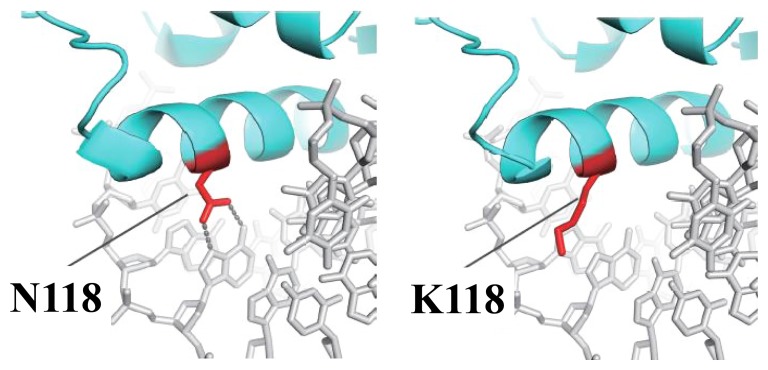
*In silico* DNA binding analysis for c.354C>G (N118K) mutation. The structural change of the mutant protein and its effect on DNA binding was assessed using a FOXK2 protein. Hydrogen bonds (dot line) between DNA and the protein were predicted to be lost due to the N118K mutation. Green line: FOXK2 protein (Thick band indicates forkhead domain). Red line: 118th amino acid of FOXK2 protein and its side chains. Gray and white lines: DNA.

Familiy 2 (**Fig. 2b**) had a c.354C>G (p.N118K) heterozygous mutation (**Fig. 3b**), which was also not found in any database and in the general population of 100 Japanese people. N118 is located in the forkhead domain of the FOXC2 protein. We investigated the effect of N118K by using FoldX. The interaction energy ΔΔG of the mutation was 1.28±0.40 kcal/mol. This finding indicated that a hydrogen bond between the side chain of N118 and the adenine base of DNA is lost, suggesting that c.354C>G is a disease-causing mutation (**Fig. 4**).

No mutation was found in the other 7 subjects with sporadic SEDACs. Structural abnormalities such as deletion or duplication of *FOXC2* were investigated using the TaqMan copy number assay; however, no such abnormality was found (data not shown).

### Clinical features of the subjects with *FOXC2* mutations

The familial cases with *FOXC2* mutations were also diagnosed as LDS, since the family members had lymphedema and/or distichiasis (**Fig. 2** **and** **Table 1**). However, the penetrance of the LDS features was incomplete. While *FOXC2* mutations were found in a total of 13 subjects, lymphedema was found in 4 and distichiasis in 9 subjects. Only 3 SEDAC subjects had both lymphedema and distichiasis. Three subjects had no SEDAC and 4 subjects had only SEDAC. SEDAC subjects without *FOXC2* mutations had no features of LDS.

**Table 1:** Clinical data of spinal extradural arachnoid cyst subjects.

Subject ID	Age at diagnosis (years)	Sex	Cyst		Surgery	Associated feature	FOXC2 mutation
			Number	Location			
*Family 1* a							
II-6	38	F	1	T9	−	Distichiasis, Lymphedema	c.733delG
III-1	15	M	3	T11-S1	+	Distichiasis	c.733delG
III-2	13	F	3	T5-10/L4-5	+	Distichiasis	c.733delG
III-5	12	F	3	T5-12	−	−	c.733delG
III-6	7	F	3	T7-10/L4-S2	−	Distichiasis	c.733delG
III-7	10	M	1	T8-9	−	−	c.733delG
III-8	7	F	1	T2-5	−	−	c.733delG
*Family 2* a							
II-1	51	F	3	T3/T4-7	+	Distichiasis, Lymphedema	c.354C>G
II-2	50	M	4	T3-8/L1	−	−	c.354C>G
III-2	29	M	5	T4-7/T11-L1/L2-L5	−	Distichiasis, Lymphedema	c.354C>G
*Sporadic*							
S-1	64	M	1	T12-L2	−	−	−
S-2	36	M	1	T11-L3	+	−	−
S-3	45	M	1	T12-L2	+	−	−
S-4	60	F	1	T12-L2	+	−	−
S-5	50	F	1	T12-L2	+	−	−
S-6	38	M	1	T12-L1	+	−	−
S-7	45	F	1	T11-L3	+	−	−

a Roman numbers represent the family numbers in the pedigrees (Fig. 2).

The age of diagnosis of the SEDAC patients with *FOXC2* mutations was significantly lower than those without *FOXC2* mutations (**Table 1**): the mean ages of SEDAC subjects with and without *FOXC2* mutations were 23.2 (range: 7–51) and 48.3 (range: 36–64) years, respectively. Male/female ratios of SEDAC with and without *FOXC2* mutations were 4/6 and 4/3, respectively. Furthermore, the number and location of the cysts were different. All SEDAC subjects without *FOXC2* mutations had a single cyst, which occurred in the thoracolumbar junction, whereas 7 of 10 SEDAC subjects with *FOXC2* mutations had multiple cysts that occurred in various areas, ranging from the upper thoracic to the sacral regions.

### Analysis of *FOXC2* mutations *in vitro*

**Figure 5:**
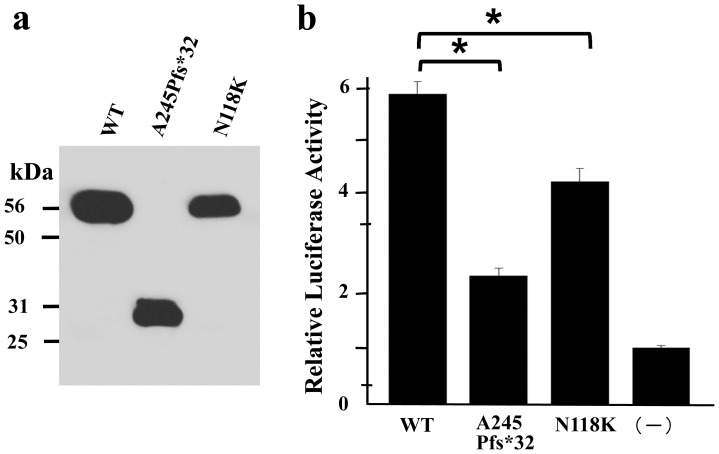
Loss of function of the *FOXC2* mutations. a) Western blot analysis. Transfected COS-7 cells were resolved by SDS-PAGE and detected by immunoblot for the N-terminal FLAG epitope. As expected, the wild-type (WT) and N118K FOXC2 proteins were 56 kDa in size, while A245Pfs*32 FOXC2 protein was 31 kDa in size. b) Dual luciferase assay. Luciferase activities of WT, A245Pfs*32 and N118K FOXC2 vectors compared with that of the empty vector (-). The significance was analysed with t-test. The activities of A245Pfs*32 and N118K FOXC2 had significantly decreased compared to that of WT FOXC2. Thick bar: mean value, error bar: SD, asterisk: p value<0.01.

COS-7 cells were transfected with vectors encoding the wild-type and mutant *FOXC2*. Whole cells were resolved by SDS-PAGE and subjected to immunoblot analysis. Detection of the N-terminal vector-encoded FLAG epitope demonstrated a stable product, 56 kDa in size, for both wild type and c.354C>G *FOXC2*. It also demonstrated a product, 31 kDa in size, for the c.733delG *FOXC2* protein (**Fig. 5a**). The effect of each *FOXC2*-pFLAG construct, on the ability to drive transactivation was tested in HeLa cells. The *FOXC2* constructs with c.733delG and c.354C>G mutations had reduced transactivation activities of 68% and 42%, respectively, when compared to the wild type (**Fig. 5b**).

## Discussion

We identified 2 novel *FOXC2* mutations in 2 familial SEDACs. To our knowledge, only 1 study on genetic analysis of SEDAC has been reported previously.[8] Our study reinforced the hypothesis that familial SEDAC is caused by *FOXC2* mutations. The *FOXC2* mutations in the family studies co-segregated with LDS, but not with SEDAC alone. The *FOXC2* mutation was also found in family members who had no SEDAC but had lymphedema or distichiasis; the penetrance of SEDAC was 70%; distichiasis, 70%; and lymphedema, 20% (**Fig. 2**). All previously reported familial cases of SEDAC have been associated with LDS.[6]-[8] However, *FOXC2* was analysed in only 1 pedigree.[8] Sanchez-Carpintero *et al.* examined a family of 48 members and identified a *FOXC2* c.298C>T (p.Q100X) mutation in 12 of them. This mutation co-segregated with LDS: distichiasis, lymphedema and SEDAC were observed in 12, 11, and 7 of the 12 subjects, respectively. Taken together, these data suggest that familial SEDAC occurs as a feature of LDS caused by *FOXC2* mutation, although symptomatic SEDAC is a rare complication of LDS.[20], [21]

The diagnosis of LDS in our subjects was difficult in the absence of any family history, since its major features have low penetrance. Four among 13 subjects with *FOXC2* mutations presented with SEDAC alone and only 3 subjects with SEDAC had both of the 2 major features of LDS (lymphedema and distichiasis). Consequently, the mode of inheritance of the disease in the family was sometimes unclear (**Fig. 2a**). Indeed, we were able to infer an autosomal dominant mode of inheritance in Family 1 only by viewing the pedigree as LDS associated with SEDAC, and not SEDAC alone. In contrast, a previous study described that the penetrance of distichiasis was 100%; lymphedema, 92%; and SEDAC, 58%. It also reported that there were no SEDAC-only subjects.[8] The difference in the penetrance of these features may be due to the variable expressivity of LDS or due to differences in the effect of the *FOXC2* mutation on the different features. In addition, these features are difficult to detect in the early stage of life. SEDAC has no symptoms in the early stage and can be diagnosed only by using MRI. Distichiasis usually has no symptoms and tends to be missed unless physicians examine the eyelids of patients carefully with this possibility in mind. Lymphedema is not usually apparent before adolescence and is not specific to LDS. Therefore, SEDAC associated with LDS is likely to be diagnosed as a sporadic SEDAC.

We showed that the *FOXC2* mutation is one of the etiological factors of SEDAC. *FOXC2* encodes a regulatory transcription factor and plays a role in the development of the mesodermal mesenchyme.[22], [23] Mice with heterozygous *Foxc2* deficiency uniformly display distichiasis and also exhibit hyperplasia, as well as incomplete valve formation in their lymphatic vessels.[24] Heterozygous *FOXC2* loss-of-function mutations like those in our subjects have been found in a number of LDS subjects.[17], [25]-[31] These findings indicate that *FOXC2* haploinsufficiency is the disease-causing mechanism of LDS. Notably, SEDAC has not been reported in the *Foxc2* heterozygous deficient mice.[24], [32]. It is not found even in *Foxc2* homozygous deficient mice, although these mice show overt spinal abnormalities.[23] However, we have a theory to explain the occurrence of SEDAC as a result of *FOXC2* mutation. *FOXC2* is expressed in the developing mesodermal mesenchyme. The dura mater originates from the mesoderm,[22], [23] and small defects in the dura mater play an important role in SEDAC pathology.[5] We propose that dura mater development is inhibited by *FOXC2* mutations in the foetus, and that as a result, the arachnoid mater protrudes from the dural defect, forming an expanding cyst.

Our study suggests that additional etiological factors of SEDAC, other than the *FOXC2* mutation, may exist because most of the sporadic subjects we studied had no *FOXC2* mutation. Interestingly, the 2 groups of SEDAC cases, with and without *FOXC2* mutations, had significantly different clinical features (**Table 1**). The age at diagnosis was significantly younger in SEDAC cases with a *FOXC2* mutation. The location of cysts was different between the 2 groups; SEDAC without *FOXC2* mutations occurred in the thoracolumbar junction while SEDAC with *FOXC2* mutations occurred at various sites ranging from the upper thoracic to sacral areas. Furthermore, there were differences in the cyst numbers; all SEDAC subjects without *FOXC2* mutations had a single cyst, while SEDAC subjects with *FOXC2* mutations frequently had multiple cysts. These data indicate that there could be other SEDAC causal gene(s). The identification of new SEDAC associated genes will help not only in early detection but also in clarifying the pathology of SEDAC.

In summary, we showed that SEDAC is caused by heterozygous loss-of-function mutations in *FOXC2*. As a result of our study, SEDAC patients and their family members will be able to use genetic screening to evaluate their risk and undergo close examination and surgery before developing irreversible neurological defects. It is easy to screen for the *FOXC2* mutation since *FOXC2* is a small 1-exon gene that can be covered by a single PCR reaction. To date, we have observed that SEDAC caused by *FOXC2* mutations is also associated with LDS. Hence, examination of family history and clinical investigation for the features of LDS are important for the early detection of the disease. However, most cases of SEDAC are not caused by the *FOXC2* mutation and show different clinical features from those with *FOXC2* mutations. Further studies aimed at identifying new SEDAC causal gene(s) are necessary to clarify their etiological factors and pathogenesis.
